# Eco-friendly lignin nanoparticles as antioxidant and antimicrobial material for enhanced textile production

**DOI:** 10.1038/s41598-024-67449-0

**Published:** 2024-07-29

**Authors:** Mohamed Abdel-Shakur Ali, Nadia Mohamed Abdel-Moein, Amal Saber Owis, Shaimaa Elsayed Ahmed, Eman Ahmed Hanafy

**Affiliations:** 1 Biochemistry Department, Faculty of Agriculture, Cairo University, Giza, Egypt; 2 Agricultural Research Center, Cotton Research Institute, Giza, Egypt

**Keywords:** Egyptian cotton, Textile, Lignin nanoparticles, Characterization, Antioxidant, Antimicrobial, Biochemistry, Microbiology, Nanoscience and technology

## Abstract

Natural polymers are bioactive compounds that are used in the treatment of several disorders. Natural lignin, an amorphous polymer, offers significant potential for use as a building block in the production of bio-renovation materials. This study used an alkaline solvent technique to extract lignin from two Egyptian cotton cultivar byproducts, Giza 86 and 90. We then created nano-lignin to recycle cotton stalks into an environmentally beneficial product. The characterization of L86, L90, LNP86, and LNP90 was carried out using particle size, zeta potential, FT-IR, and TEM. Antioxidant activity using the DPPH assay and antimicrobial activity were determined for lignin and nano-lignin. Seven pathogenic bacteria (*Bacillus cereus*, *Staphylococcus aureus*, *Staphylococcus sciuri*, *Salmonella typhi*, *Salmonella enterica*, *Escherichia coli*, and *Pseudomonas aeruginosa*) and five mycotoxigenic fungi (*Aspergillus flavus*, *Aspergillus ochraceus*, *Aspergillus niger*, *Fusarium proliferatum* and *Penicillium verrucosum*) were used for antimicrobial activity. The results showed high antioxidant efficiency for LNP90, with an IC_50_ of 10.38 µg/mL. The antimicrobial activity showed positive growth inhibition for all studied microorganisms, with significant differences in nano-lignin compared to ordinary lignin. lignin and nano-lignin were effectively applied to treated textiles for medical purposes. The study concluded that single-use medical textiles with anti-microbial and anti-oxidant properties, made from lignin and nano-lignin, could benefit patients intolerant to antibiotics.

## Introduction

Egyptian cotton (*Gossypium barbadensis*) is the finest cotton known because it possesses a few noble qualities that set it apart from other natural fibers^1^. The cotton stalk is Egypt’s most significant agricultural byproduct. It can be identified by possessing branches, carrying leaves that are not open and continue to root, and being hazardous to the environment if burned or transported with cotton bollworm larvae and weed seeds^2,3^.

Lignin is the second-largest lignocellulosic biopolymer (after cellulose) on earth^4^ and one of the main components of the plant cell wall^5^. Three phenylpropane monomers make up lignin’s three-dimensional network structure. They are para-coumaryl alcohol, sinapyl alcohol, and coniferyl alcohol. These are linked together by carbon–carbon and ether linkages^6^. A variety of functional groups differentiate the aromatic ring that makes up the polymeric structure of lignin. It contains aliphatic and methoxyl groups, carboxylic, carbonyl, and phenolic hydroxyl groups, as well as a propanoid chain. It functions as a physical barrier to stop the spread of diseases and their toxins because of its heterogeneity, hydrophobic nature, and insolubility in aqueous environments^7^. As a result, efforts are being made in both research and industry to use lignin in the manufacturing of polymeric drug encapsulation and scaffold materials^8^. The lignin’s physical and chemical behavior will be different according to the plant source and extraction conditions, temperature, and residue/solvent ratio due to variations in the basic compound of lignin^9,10^.

The use of traditional polymeric materials offers numerous benefits. Although their resistance to biological agents is harmful to the environment, the increasing interest in organic recycling, sustainability, environmental challenges, and healthcare should lead to the widespread use of biodegradable polymers^11^. Cleaner technology techniques using biodegradable materials encourage global pollution control. Cleaner production techniques that make use of natural, sustainable, and safe resources decrease the damaging effects that conventional manufacturing industries’ operations and byproducts have on the environment. Various contexts apply natural substitutes with specific benefits, such as plant proteins, cellulose, polylactic acid, polyhydroxy butyrate, starch, and bio-based polyamide. Numerous fields have thoroughly tested and applied biopolymer-based products^12^.

Nanotechnology, an extremely small form of technology that uses the nanometer scale, discusses the practical application of materials, mechanisms, devices, and systems at a nanoscale. Nanotechnology is a multidisciplinary field of research. Nanotechnology is being used in a wide range of industries, and it is expected that it will open up a wide range of new opportunities for innovation and contribute to the advancement of many scientific subjects. Particularly, it is anticipated that these developments will occur in several biomedical applications, including drug delivery, molecular imaging, biomarkers, biosensors, and numerous other applications^13–15^. The spherical form of nano-lignin is generally considered to have the lowest toxicity level, and its nanoparticles are completely biodegradable^16,17^. Lignin is cheaper than silver nanoparticles by approximately 1,500 times. This shows the potential of utilizing lignin as an antimicrobial agent as a replacement for traditional metallic antimicrobial compounds^18^. Nano-lignin has been produced using an ultrasonic process^10^. By adjusting the ultrasonic treatment period and combining the excellent colloidal stability of the resulting dispersion with a waterborne thermoplastic polyurethane matrix, it was possible to significantly reduce the size of the lignin particle to approximately 10–50 nm^19^. The physical process of converting alkali lignin into nanoparticles through ultrasonic irradiation reveals that the type of lignin determines the compositional and structural changes of the resulting nanoparticles, rather than the intensity employed significantly altering them^20^. Lignin nanoparticles are getting more and more attention because they are biodegradable and biocompatible and can fight free radicals, bacteria, or UV light. Biomedicine, adsorbent materials, nanocarriers, medication release, and environmental restoration widely utilize lignin nanoparticles as great alternatives to some partially harmful nanomaterials^10^.

Compared to other materials such as organics, ceramics, and glasses, the biodegradable polymer maintains cell homeostasis and improves cell-matrix interaction and cell signaling pathways. For a biomedical device to interact with a patient's body in a more biocompatible way, its chemical functions and qualities are crucial. According to Zhang et al.^21^, biodegradable polymeric macromolecules have recently gained greater potential benefits over synthetic inorganic biomaterials in the biomedical area in terms of half-life, stability, safety, and ease of manufacture. Lignin is a suitable precursor for the production of environmentally friendly nanoparticles because it is biodegradable, biocompatible, and displays extremely good stability^22^. However, due to its properties, such as its complicated macromolecular structure that depends on its botanic origin, extraction method, and significant molecular differences within the same batch, lignin is a difficult starting material to work with^23,24^.

## Materials and methods

### Chemicals and microorganisms

All chemicals and reagents were purchased from Sigma-Aldrich Co. (Poole, London, England). Different types of foodborne pathogenic bacteria and mycotoxigenic fungus species such as *Staphylococcus aureus* (ATCC 13565), *Staphylococcus sciuri* (2–6), *Bacillus cereus* (EMCC 1080), *Salmonella enterica* (SA19992307), *Salmonella typhi* (ATCC 25566), *Escherichia coli* (0157 H7 ATCC 51659), *Pseudomonas aeruginosa* (NRRL B-272), *Aspergillus flavus* (NRR 3357), *Aspergillus ochraceus* (ITAL 14), *Aspergillus niger* (IMI 288550), *Fusarium proliferatum* (MPVP 328), and *Penicillium verrucosum* (BFE 500) were obtained from National Research Centre, Giza, Egypt.

### Preparation of the cotton stalk samples

The Giza Agricultural Research Station, cotton research institute (CRI), and agricultural research centre in Egypt (ARC) planted two Egyptian cotton cultivars, Giza 86 and 90, in a field trial experiment. The cotton stalks were gathered and stored after picking the cotton fiber. Following a two-week drying period, the cotton stalks from the two cultivars under investigation were cut into 2.5–4 cm-long splinters. After that, we ground the samples using a 0.4-mm screen in a wood crusher. Before being analyzed, they were ground one more time in a powerful mixture mill.

### Extraction of lignin by a modified steam explosion process

Lignin extractions from the two cotton stalk cultivars Giza 86 and Giza 90 (L86 and L90) were conducted according to Ibrahim et al*.*^25^ with some modifications. About 10 g of milled cotton stalks were steamed on a specific explosion system for one hour. The steamed fibers were extracted with water at 80ºC for one hour, then filtered and washed with 500 ml of water, followed by further extraction with 20% sodium hydroxide (NaOH) at 80 °C in a fiber-to-liquid ratio of 1:10 for one hour, then filtrated and the solution acidified to pH 1.5 by sulfuric acid. The solution was incubated in a water bath at 100 °C for one hour with continuous stirring. Lignin was washed with hot water until neutral, then dissolved in 17.5 (w/w) NaOH. Lignin was precipitated as previously and washed again with hot water until the filtrate became colorless.

### Nano-lignin preparation (LN86 and LN90)

With slight modifications, nano-lignin was created using the method described by Gilca et al*.*^26^. Aqueous lignin suspensions (0.7%) were treated to create nano-lignin using an ultrasonic horn (Sonics and Materials VC600/CV17) with 600 W of power and 20 kHz of frequency for 60 min. Giza 86 and 90 nano-lignin were produced by carefully controlling the drying process of the nano-lignin.

### Characterization of L86, L90, LNP86, and LNP90

#### Particle size (PS) and zeta potential (ZP)

In the pharmacy faculty’s central lab at Cairo University, a Zetasizer 3000 particulate size description analyzer (Malvern Instruments) was used to measure the PS of L86, L90, LNP86, and LNP90, as well as the ZP of LNP86 and LNP90. At 25 °C and a 90° scattering angle, the size was measured three times, consuming three minutes for each measurement. The mean hydrodynamic diameter was ascertained using continuous analysis. The ZP was measured automatically using an aqueous dip cell^27^.

#### Fourier transform infrared spectroscopy (FTIR)

To produce incredibly small particles, anhydrous potassium bromide (KBr) was added to the samples before grinding. The granules were then crushed into two thin pellets for examination. Using a Fourier-transform infrared (FTIR) spectroscopic analyzer (Model JASCO FTIR-6100) in the scanning range of 4000–400 cm^−1^, the infrared spectra were captured. The sample’s previously recorded spectra were used to modify the spectra’s baseline, and three or five points were added to the spectra to smooth them out^27^.

#### Transmission *electron* microscopy (TEM)

With a transmission electron microscope (JEM-1400, JEOL model), the morphological screening of nano-lignin was performed. Image processing was carried out at the electronic microscopy lab at Cairo University Research Park (CURP). A glow-discharged carbon grid was used to hold freshly made Ag-NPs, and they were given a brief amount of time to air dry. Next, utilize a TEM to check the shape and surface roughness of the nanocomposite specimens^3^.

### Radical scavenging activity of L86, L90, LNP86, and LNP90 against DPPH assay

The Wright et al*.*^27^ technique was slightly modified to evaluate the effectiveness of L86, L90, LNP86, and LNP90 at scavenging 2,2-diphenyl-1-picrylhydrazyl radicals (DPPH). Many serial concentrations of samples were prepared in small test tubes (12.5, 25, 50, and 100 μL, respectively). 0.5 mL of methanol was added after thoroughly vortexing each test tube. Then, 0.5 mL of a 0.1 mM methanolic solution of DPPH was added. The test tubes were vortexed once more, and then they were all left to stand in the dark for 30 min at room temperature. A spectrophotometer was employed to determine the yellow color that, compared with the blank reagent, was at a wavelength of 517 nm. As a standard antioxidant, gallic acid was used. The following equation was used to calculate the ability to scavenge DPPH radicals^28^:

$$ {\text{DPPH scavenging inhibition }}\left( \% \right) \, = \, \left[ {\left( {{\text{Ac}} - {\text{As}}} \right)/{\text{Ac}}} \right] \, \times { 1}00 $$

where Ac is negative control absorption and As is sample absorption.

### Antimicrobial activity of L86, L90, LNP86, and LNP90

The antibacterial properties of L86, L90, LNP86, and LNP90 were tested on seven different types of foodborne pathogenic bacteria. Three gram-positive bacteria, and four gram-negative bacteria, Stock cultures were grown for 24 h at 37 °C on slant nutrient agar, after which they were kept in the fridge until required. The five mycotoxigenic fungus species used in the antifungal test were kept on slant potato dextrose agar (PDA) at 25 °C in a refrigerator until further use.

#### Broth dilution technique

The disc diffusion method was used, according to Bauer et al*.*^29^, to measure the reactivity of lignin and nano-lignin in different bacterial cultures. Four to five milliliters of tryptic soy broth (TSB) were poured into a tube along with a loop full of nutritional agar slant for each species of bacteria. A spectrophotometer was used to measure the standard turbidity density at 625 nm after the TSB had been incubated at 35 °C for 2 to 6 h. Cotton swabs were used for transferring bacterial cultures from TSB to a Petri plate that had been prepared with 20 mL of nutritional agar. Sterile forceps were used to place the discs on the ready-made Petri dish. A positive control of tetracycline (500 ppm) and a negative control of DMSO had been used. The diameter of the disc and the inhibition zone (mm) of free growth were measured following a 24 h incubation period at 37 °C.

Petri plates containing Yeast Extract with Supplements (YES) media were infected with 0.05 mL of each fungal culture, and the inoculum was equally distributed using a sterile L-glass rod. Fungal strains were cultured on potato dextrose agar (PDA) at 25 °C for five days. The loaded discs containing lignin and lignin nanoparticle extracts were placed on the seeded plates. DMSO was used as a negative control, while the commercial fungicide Nystatine (1000 units/mL) was employed as a positive control. For the examined fungus, the inhibitory zone (mm) was measured. Before calculating averages, three replicates of each treatment were carried out^30^.

#### Minimum inhibitory concentration (MIC) determination

Using the Wiegand et al*.*^31^ tube dilution approach, the minimum inhibitory concentrations (MIC) of extract that inhibited the development of the test organism was determined. The examined bacteria were diluted in a 10 mL culture tube with tryptic soy broth with the 0.5 McFarland standard to obtain 108 CFU mL^−1^ inoculation. DMSO was used to generate extracts of lignin and nano-lignin in ten different concentrations (5, 2.5, 1.5, 1.0, 0.75, 0.50, 0.25, 0.1, 0.05, and 0.01 mg/mL). When the suspension was inoculated with 100 μl of bacteria and left to sit at 37 °C for a day, growth became apparent as turbidity.

The MIC of lignin and lignin nanoparticle extracts at 10 concentrations (5.0, 2.5, 1.5, 1.0, 0.75, 0.50, 0.25, 0.1, 0.05, and 0.01 mg/mL) was evaluated for the fungus according to Sokmen et al*.*^32^. Each of them was dissolved in 0.5 mL of 0.1% Tween 80 and 9.5 mL of PDA before being put into a 6 cm Petri dish. Using a 3 μL fungal suspension containing 108 CFU mL^−1^ and 0.5 McFarland standard, the plates were centrally inoculated. The plates were incubated for 24 to 48 h at 25 °C.

### Application of lignin and nano-lignin on textiles

We evaluated the antibacterial activity of lignin and nano-lignin to make medical gauze fabric more cost-effective. The tests were made against three pathogenic gram-positive bacteria: *Staphylococcus aureus* ATCC 13565, *Staphylococcus sciuri* 2–6, *Bacillus cereus* EMCC 1080, and two pathogenic gram-negative bacteria: *Salmonella enterica* SA19992307, Salmonella typhi ATCC 25566, Escherichia coli 0157 H7 ATCC 51659, and *Pseudomonas aeruginosa* NRRL B-272, as described in Bauer et al*.*^29^. Five species of mycotoxigenic fungi, as described in Medeiros et al*.*^30^, *Aspergillus flavus* NRR 3357, *Aspergillus ochraceus* ITAL 14, *Aspergillus niger* IMI 288550, *Fusarium proliferatum* MPVP 328, and *Penicillium verrucosum* BFE 500, were also employed for antimicrobial activity tests.

### Statistical analysis

Chemical assays were carried out in triplicate, and mean standard error were used to represent the results. The statistical analysis was done using the M-state program version 2.0, according to Tishkovskaya and Lancaster^33^. Randomized complete block design (RCBD) analysis in a factorial way was used, followed by Fisher’s LSD (least significant differences) technique (≥ 0.05) for comparing significant changes between treatments.

## Results and discussion

### Characterization of L86, L90, LNP86, and LNP90

#### Particle size analysis (PS)

**Figure 1:**
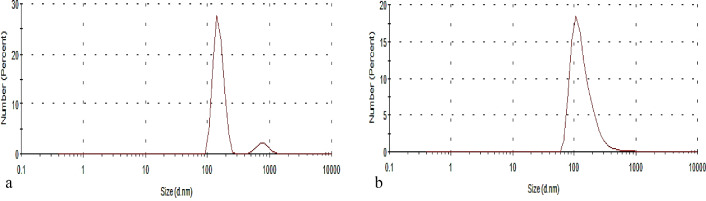
Size distribution by number of (**a**) L86 and (**b**) LNP86.

**Figure 2:**
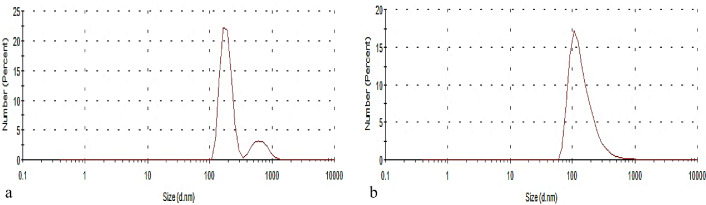
Size distribution by number of (**a**) L90 and (**b**) LNP90.

Figures 1, 2 illustrate the light scattering profiles of lignin suspensions of L86, L90, LNP86, and LNP90 before and after ultrasound treatment. The average particle diameter decreases significantly in each scenario, from 780–648 to 143–151 nm. The polydispersity index (PDI) of L86 and L90 ranged from 0.778 to 0.812, indicating the broad size distribution and presence of large particles. The PDI values of LNP86 and LNP90 were 0.355 and 0.327, respectively. According to Zielińska^34^, the increase in the surfactant concentration decreased the PDI to values lower than 0.4, which proves the formation of the nanoparticles. Therefore, the ultrasonic technique successfully transformed lignin into nanoparticles. These findings are in agreement with those from Ali et al*.*^2^, Gupta et al*.*^11^, Li et al*.*^35^, Lievonen et al*.*^36^, Chen et al*.*^37^, and Rahman et al*.*^38^.

#### Zeta potential distribution (ZP)

**Figure 3:**
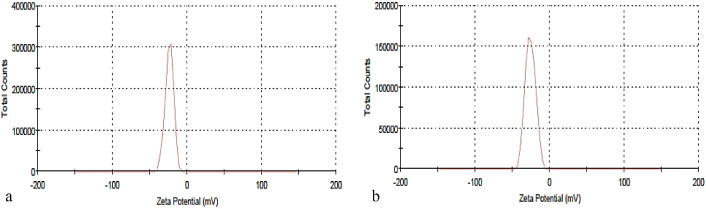
Zeta potential of (**a**) LNP86 and (**b**) LNP90.

Zeta potential assay demonstrated the number of hydroxyl groups in the aromatic ring and ortho substitutions with the electron-donating methoxy groups. The results in (Fig. 3a,b) demonstrated that the surfaces of LNP86 and LNP90 contain a negative charge of approximately −23.8 and −25.8 mV, respectively. The relatively high negative zeta potential keeps the particles from sticking together and creates strong electrical double-layer repulsion. This lets the nanoparticles’ zeta potential signal the electrical surface properties of the particle. So there was no particular aggregation, and the lignin nanoparticle dispersion was very stable in pure water. These findings are consistent with those of Lievonen et al*.*^36^, Jiang^39^, and Beisl et al*.*^40^.

#### Fourier transform infrared spectroscopy (FT-IR)

**Figure 4:**
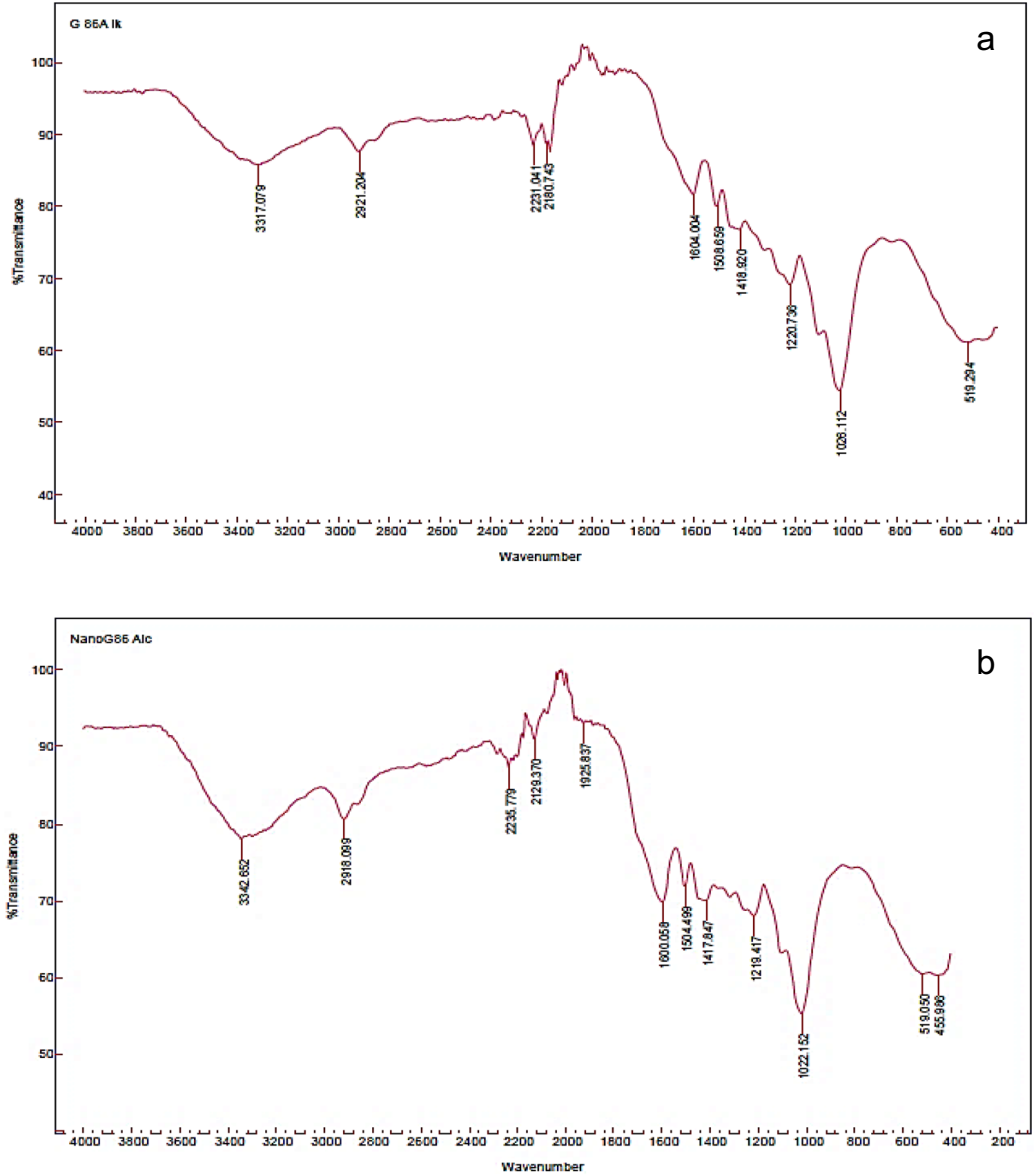
FT-IR spectra of (**a**) LNP86 and (**b**) L86.

**Figure 5:**
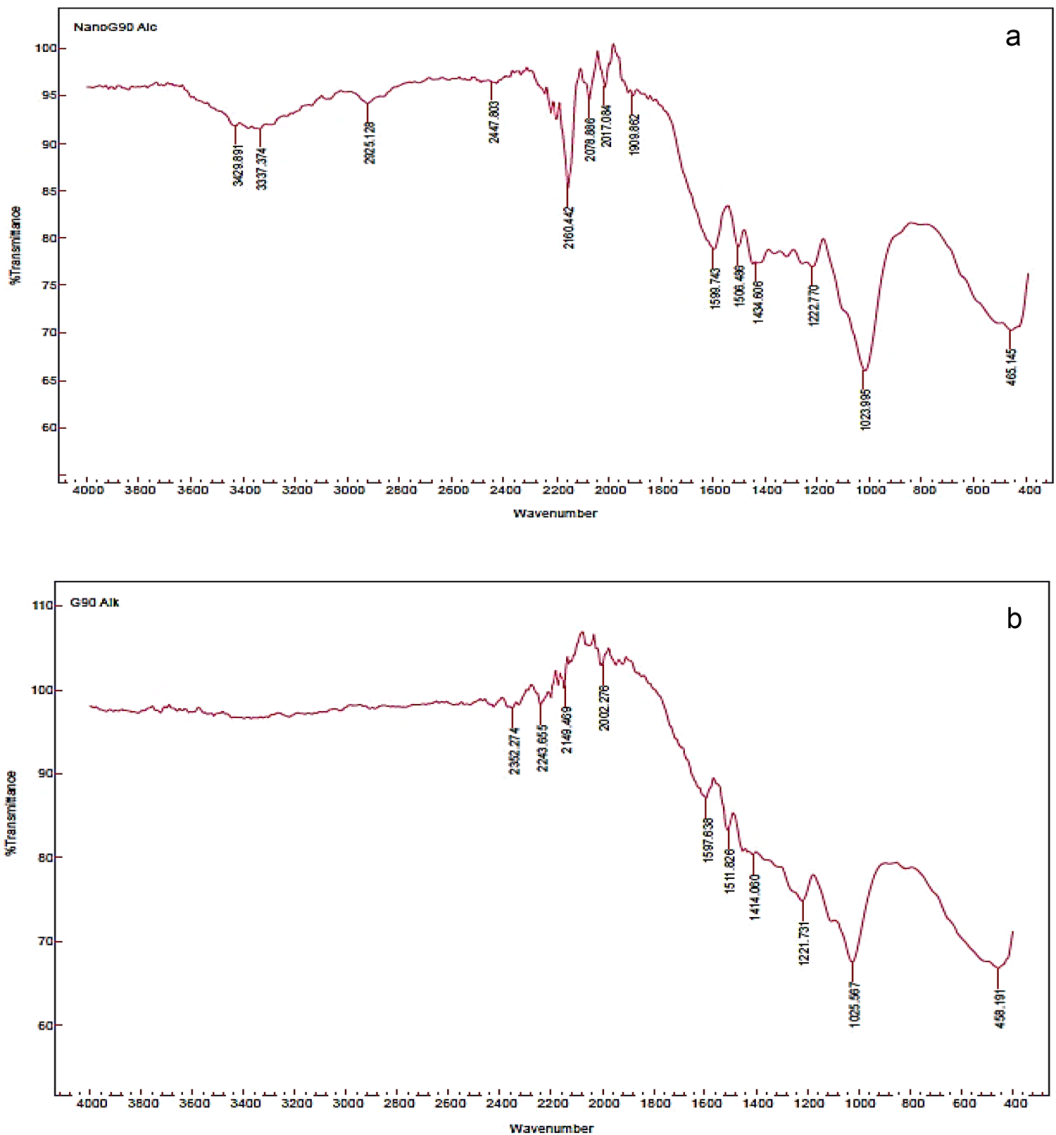
FT-IR spectra of (**a**) LNP90 and (**b**) L90.

FT-IR analysis greatly aids the structural investigation of lignin and nano-lignin, as well as research into their antioxidant and antibacterial properties and associated structure–property connections. According to the information shown in (Figs. 4, 5a,b), the typical bands of absorption for the aromatic structures of LNP86, L86, LNP90 and, L90, respectively, ranged from 494 to 3907 cm^−1^ in the FT-IR spectrum. It is important to note that the following bands were present in all samples: 1129–1180 cm^−1^ (was C = C); 1417–1434 cm^−1^ (was CH bonds of OCH groups); 1505–1604 cm^−1^ (was C = C aromatic ring); 2913–2921 cm^−1^ (was aromatic ring OCH_3_); and 3317–3429 cm^−1^ (was OH alcohols, phenols). These results are close to the findings of Gilca et al*.*^26^, and Kang et al*.*^41^. On the other hand, LNP86 to L86 showed some new bands arose in nano states, such as 455 cm^−1^ (with tri-substituted alkenes) and 1925 cm^−1^ (with C-H aromatic compound bending), and when comparing LNP90 to L90, the spectra indicated that nano-state was excellent by the following bands: 1909–2017, 2078, 2447, 2925, 3337, and 3429 cm^−1^ with (C = C = C allene stretching, C=C conjugated, O–H stretching in carboxylic acid, Aromatic OCH_3_, O–H alcohols, phenols, and O–H stretching intermolecular bonded, respectively). The spectra showed that L90 was better than L86 in the active groups that support antioxidant properties, like phenolic O–H, and also make them more antimicrobial, like phenolic O–H, O–CH3, and C=C. The results were consistent with those found by Poletto and Zattera^42^, as well as Ponomarenko et al*.*^43^. Additionally, the spectrum showed a strong OH band, either introduced to the lignin during the alkali treatment or contributed by the incomplete removal of hemicellulose during the fractionation process. Hemicellulose frequently contains pentose and hexose units, which explains the presence of C–O–C. The outcomes agreed with those reported by Ali et al.^2^, and Ab Rahim et al.^44^.

#### Transmission electron microscopy (TEM)

**Figure 6:**
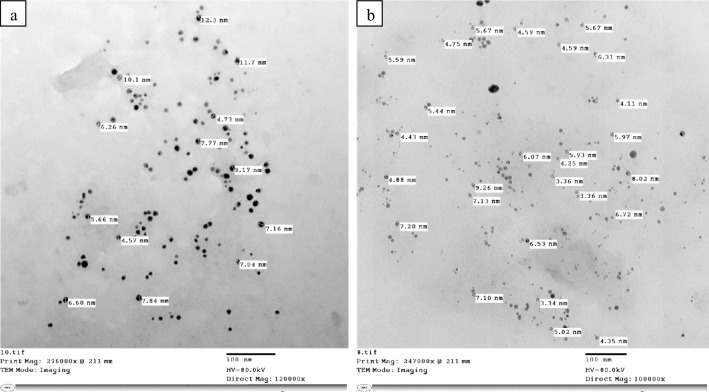
Electron micrograph of (**a**) LNP86 and (**b**) LNP90*.*

Figure 6(a,b) represent the surface morphology of LNP86 and LNP90, as well as the TEM of these materials, which had a size range of 3.34–12.3 nm and were spherical. The result supported the aforementioned zetasizer assay data regarding the nanoscale of lignin. These findings agreed with the results obtained by Ali et al*.*^2^, Gupta et al*.*^16^, Rahman et al*.*^38^, and Mishra and Ekielski^45^**.**

### Radical scavenging activity of L86, L90, LNP86, and LNP90 against DPPH

A spectrophotometer was used to measure the antioxidant activity because DPPH is a spectrophotometric transfer-based electron test that evaluates an antioxidant's capacity to lower an oxidant, which changes colour when reduced. The amount of colour change correlates to the number of antioxidants present in the sample. According to the current investigation, L86, L90, LNP86, and LNP90 exhibit some free radical scavenging properties. According to the study, the amount of DPPH radical scavenging activity developed as the concentration increased for different lignin samples. The most effective radical scavenger was LNP90, with an IC_50_ of 10.38 μg/mL. According to Table 1, L90 had the highest antioxidant activity (84.492.1%) at 100 g/mL, while LNP86 had the lowest antioxidant activity (45.312.6%) at 25 g/mL. According to research by Ali et al*.*^2^, Figueiredo et al*.*^46^, and Gao and Fatehi^47^, functional groups like methoxy and phenolic hydroxyl groups in lignin can stop oxidative propagation processes through hydrogen donation. Due to its increased solubility in water, the prepared LNP90 exhibited stronger antioxidant activity as well as enhanced DPPH radical scavenging, superoxide radical scavenging, and reducing power^48,49^.

**Table 1:** DPPH Scavenging radicals (%) and IC_50_ of antioxidant activity of LNP86, LNP90, L86 and L90.

DPPH scavenging radicals % (mean ± S.E.)					
	Concentration (ppm)				IC_50 (_µg/mL)
	12.5	25	50	100	
L86	55.79 ± 1.4^de^	63.50 ± 5.1^d^	66.93 ± 2.8^d^	69.00^e^ ± 0.32e	11.31^c^
L90	48.95 ± 0.72^ef^	56.00 ± 3.6^e^	68.00 ± 1.4^d^	84.49^c^ ± 2.1c	19.30^b^
LNP86	45.3 ± 2.6f.	52.52 ± 3.5^e^	59.57 ± 5.3^e^	66.32^e^ ± 1.3e	24.93^a^
LNP90	60.88 ± 0.7^bcd^	61.82 ± 1.9^d^	68.52 ± 2.1^d^	78.81^d^ ± 0.37d	10.38^c^
Gallic acid	77.7 ± 0.1^a^	83.9 ± 0.1^a^	99.0 ± 0.1^a^	99.4^a^ ± 0.1a	8.04^c^
LSD (0.05)	7.85	4.68	5.57	4.38	4.95

Each value represents the mean ± SE. Values with the same letter are not significantly different at (P ≤ 0.05).

Alkali lignin is made up of derivatives of hydroxycinnamic acid. The zeta potential assay results for LNP solutions (−25 mV) proved the antioxidant activity of hydroxycinnamic acid. All of these things, along with the fact that lignin nanoparticles are small and have a lot of surface area, make the phenyl group of lignin more capable of holding protons^50^. Finally, we can say that lignin’s complex structure, which consists of aromatic rings with hydroxy and methoxy functional groups, is what gives it its antioxidant capacity.

### Inhibition zone of L86, L90, LNP86, and LNP90 against different microbial strains

Tables 2, 3 show the results of the disc diffusion technique. All pathogens, including five fungi and seven bacteria, demonstrated lignin use (L86, L90, LNP86, and LNP90). Lignin influenced the presence of inhibitory zones in all examined pathogens. With L86 and L90, the inhibition zones for the treated fungi varied from 8.0 to 11.7 mm, and with LNP86 and LNP90, they ranged from 9.7 to 14.2 mm. The inhibition zones for the treated bacteria ranged from 9.3 to 13.7 mm for L86 and L90 and from 11.3 to 19.5 mm for LNP86 and LNP90.

**Table 2:** Inhibition zone (mm) of LNP86, LNP90, L86 and L90 against different fungal strains.

Fungi	Inhibition zone (mean ± S.E.)(mm)					
	DMSO	Nystatin	LNP86	L86	LNP90	L90
*Aspergillus flavus*	0	16.0 ± 0.76^a^	10.3 ± 0.76^c^	9.3 ± 1.04^ef^	11.2 ± 0.82^d^	10.2 ± 0.86^e^
*Aspergillus ochraceus*	0	10.8 ± 0.36^cd^	9.7 ± 0.48^de^	8.8 ± 0.76f	10.2 ± 1.25^e^	9.3 ± 0.58f
*Aspergillus niger*	0	8.8 ± 0.14f	11.2 ± 1.04^b^	9.0 ± 0.50f	11.2 ± 0.76^d^	8.5 ± 1.00^g^
*Fusarium proliferatum*	0	11.2 ± 0.58^c^	10.2 ± 0.84^cd^	8.0 ± 0.42^g^	12.5 ± 1.32^b^	8.7 ± 0.58^g^
*Penicillium verrucosum*	0	9.8 ± 1.04^def^	14.2 ± 1.04^a^	11.2 ± 1.44^b^	13.2 ± 0.76^a^	11.7 ± 1.14^c^
LSD (0.05)		1.22	0.55		0.45	

n = 3, *S.E.: standard error, different subscripts are significantly different at the 5% level, negative control: DMSO, positive control: Nystatin.

**Table 3:** Inhibition zone (mm) of LNP86, LNP90, L86, and L90) against different bacterial strains.

Bacteria	Inhibition zone (mean ± S.E.)(mm)					
	DMSO	Tetracycline	LNP86	L86	LNP90	L90
*Bacillus cereus*	0	16.6 ± 0.58d^ef^	12.0 ± 0.50^c^	9.3 ± 1.04^i^	12.2 ± 0.76^ef^	11.0 ± 0.50^gh^
*Staphylococcus aureus*	0	18.0 ± 1.04^bcd^	13.2 ± 1.04^b^	10.2 ± 0.76^gh^	12.5 ± 0.50^ef^	11.0 ± 0.50^gh^
*Staphylococcus sciuri*	0	17.7 ± 0.86^bcd^	14.8 ± 1.04^a^	12.3 ± 0.76^c^	13.0 ± 2.18^de^	10.5 ± 0.50^h^
*Escherichia coli*	0	18.8 ± 1.14^bc^	12.8 ± 1.04^b^	10.7 ± 1.04^ef^	17.2 ± 1.76^b^	13.7 ± 0.28^d^
*Salmonella typhi*	0	15.5 ± 0.50^jf^	12.2 ± 0.86^c^	10.5 ± 0.32^fg^	12.7 ± 0.76^e^	13.0 ± 0.42d^e^
*Salmonella enterica*	0	16.0 ± 1.00^ef^	11.3 ± 0.76^d^	10.0 ± 0.50^h^	16.0 ± 1.32^c^	11.7 ± 1.04^fg^
*Pseudomonas aeruginosa*	0	19.2 ± 0.58^ab^	12.2 ± 1.04^c^	11.0 ± 1.04^de^	19.5 ± 1.32^a^	12.8 ± 1.25^de^
LSD (0.05)		1.61	0.42		0.95	

n = 3, *S.E.: standard error, different subscripts are significantly different at the 5% level negative control: DMSO, positive control: Tetracycline.

L86 showed twice as much activity as Nystatin against the fungi *Penicillium verrucosum* and *Aspergillus niger*. This showed that all samples were natural, biodegradable, and safe for the environment, but they could still compete with Nystatin’s antifungal properties. We observed wider inhibition zones than the comparable L86 and L90 zones, suggesting a stronger impact of LNP86 and LNP90. With rare exceptions, the same pattern was seen for the inhibitory zone of unstudied microorganisms. The grand average comparison showed that lignin and lignin nanoparticle treatments have a greater impact on bacteria than fungi.

### MIC of L86, L90, LNP86, and LNP90 against different microbial strains

For treated fungi, the MIC determination ranged from 2.0 to 11.7 mg/mL with L86 and L90, and between 1.2 and 4.2 mg/mL with LNP86 and LNP90, as shown in Tables 4, 5. According to the concentrations of the treated bacteria, the MIC ranged between 1.2 and 6.7 mg/mL for LNP86 and LNP90, and between 3.3 and 11.0 mg/mL for L86 and L90. The MIC values for gram-positive bacteria ranged from 2.2 to 10.8 mg/mL and 1.2 to 11.0 for gram-negative bacteria, revealing that gram-negative bacteria are slightly more sensitive to lignin than positive bacteria. The sensitivity of bacteria varied according to the type of lignin used. The fungi that were particularly sensitive to lignin and nano-lignin were *Aspergillus ochraceus* and *Fusarium proliferatum*.

**Table 4:** The MIC (mg/mL) of (LNP86, LNP90, L86 and L90 against pathogenic fungi.

Fungi	MIC mg/mL (mean ± S.E)			
	LNP86	L86	LNP90	L90
*Aspergillus flavus*	4.2 ± 0.76^d^	6.7 ± 0.36^b^	4.00 ± 1.14^d^	11.7 ± 0.48^a^
*Aspergillus ochraceus*	2.2 ± 0.28f	4.2 ± 0.36^d^	3.00 ± 1.00^e^	3.3 ± 0.42^e^
*Aspergillus niger*	3.0 ± 0.76^e^	5.8 ± 0.36^c^	4.20 ± 1.14^d^	10.0 ± 0.96^b^
*Fusarium proliferatum*	1.2 ± 0.28^g^	2.0 ± 0.28f	0.80 ± 0.18f	4.2 ± 0.21^d^
*Penicillium verrucosum*	2.8 ± 1.28^e^	7.8 ± 0.28^a^	4.00 ± 1.00^d^	7.7 ± 0.58^c^
LSD (0.05)	0.57		0.65	

n = 3, *S.E.: standard error, different subscripts are significantly different at the 5% level.

**Table 5:** MIC (mg/mL) of LNP86, LNP90, L86, and L90 against pathogenic bacteria.

Bacteria	MIC mg/mL (mean ± S.E)			
	LNP86	L86	LNP90	L90
*Bacillus cereus*	4.2 ± 0.86f	9.2 ± 0.96^b^	2.2 ± 0.58f	6.7 ± 0.48^b^
*Staphylococcus aureus*	5.8 ± 1.04^e^	10.8 ± 0.42^a^	3.2 ± 1.60^e^	6.7 ± 0.96^b^
*Staphylococcus sciuri*	2.3 ± 0.28^h^	4.2 ± 0.48f	4.2 ± 1.18^d^	6.7 ± 0.48^b^
*Escherichia coli*	3.3 ± 1.04^g^	6.7 ± 0.96^d^	1.2 ± 0.28^g^	5.8 ± 0.42^c^
*Salmonella typhi*	3.2 ± 1.61^g^	6.7 ± 0.48^d^	2.2 ± 0.58f	4.2 ± 0.48^d^
*Salmonella enterica*	4.2 ± 1.44f	7.5 ± 0.83^c^	2.2 ± 0.28f	3.3 ± 0.42^e^
*Pseudomonas aeruginosa*	5.8 ± 1.04^e^	11.0 ± 0.96^a^	6.7 ± 1.46^b^	10.7 ± 1.25^a^
LSD (0.05)	0.63		0.76	

n = 3, *S.E.: standard error, different subscripts are significantly different at the 5% level.

According to Dizhbite et al*.*^51^, there is a correlation between the soluble fraction’s radical scavenger activity and Kraft lignin’s antibacterial activity. Dong et al*.*^52^ even suggested that the antioxidative and antibacterial properties of maize straw lignin extract were complementary. Cotelle et al*.*^53^ stated that lignin’s phenolic moieties, which scavenge free radicals to provide antioxidant effects, include free reactive radicals like non-etherified hydroxyl phenolic and ortho-methoxy groups. Lignin has been shown in numerous studies to inhibit the growth of different types of bacteria^54^. Rahouti et al*.*^55^ observed that certain strains exhibited physiological changes (fructification modifications, aberrant synthesis of pigments, or viscous substances) when cultivated in the presence of phenolic substrates.

Some components of lignin can inhibit the development of bacteria such as *Aspergillus niger*, and *Escherichia coli*, as well as the activity of certain enzymes. The side chain structure and functional group composition of phenolic compounds play an important role in lignin’s antibacterial activity. While functional groups containing oxygen (hydroxyl, carbonyl, and ester groups) in the side chain of phenolic components have less of an inhibitory effect, double bonds and methyl groups increase the biocidal effect of phenolic components^2^. Lignin demonstrated antimicrobial properties against yeast, gram-negative bacteria, and gram-positive bacteria^56^. Carvacrol and Thymol, two lignin phenolic monomers, have antimicrobial properties because they damage bacterial cell membranes and lyse bacteria, releasing their contents into the environment, whereas Cinnamaldehyde can penetrate bacterial cell membranes, lowering intracellular pH and depleting ATP^57^. According to Mahmood et al*.*^58^, phenolic fragments that have oxygen (-OH, -CO, and COOH) functional groups in their side chain exhibit lower inhibitory activity compared to those that have methyl groups in the γ-position and C–C double bonds in the α- and β-positions of their side chain. In comparison to unmodified lignin extracts, the antibacterial activity was enhanced by chemically modifying functional groups, such as by adding epoxy groups to lignin extracts^59^. So lignin contains antimicrobial agents and antioxidant monomers, making it capable of playing a role in the food industry, improving healthcare, agriculture, animal welfare, ethanol production, and green bio-oil^18,60^.

The phenolic compounds' side chain structure and functional group play a significant role in determining the antioxidant and antimicrobial properties of lignin. The extraction process of lignin nanoparticles (LNPs) can impact the final product's molecular weight, purity, and chemical structures. Enhancing the behaviour of the ant microorganism with higher purity, a larger surface area, and a smaller LNP would be more beneficial than a pristine one^50^**.** Polyphenols first lyse the cell wall, which effectively releases the intracellular fluid. It was assumed that a large number of reactive oxygen species (ROS) were accumulated on the surface of LNP because of its strong anti-oxidation behaviour. When these ROS interact with bacteria, they alter the normal redox physiological process, potentially leading to the release and induction of oxidative stress. This discovery suggests that lignin’s antioxidant activity and its antibacterial properties are synchronised through the formation of ROS^61^. Additionally, due to their small size, nanoparticles may cross the cell barrier and enter the bacterial cell. During this process, some lignin-derived monophenolic chemicals, such as cinnamaldehyde, can pass through the bacterial cell membrane, lowering the intracellular pH and depleting ATP^62^**.** We thus conclude that the antimicrobial mechanism by which lignin inhibits microbial growth may be caused by the presence of various reactive oxygen species in addition to the chemical structure of lignin’s adhesion to the microbial cell membrane.

### Application of lignin and nano-lignin on textiles

Particularly for patients who are resistant to antibiotics and suffer from conditions like diabetes, hypertension, and renal failure, nano-lignin and separated lignin were investigated as new materials for single-use clinical textiles. Table 6 displays the inhibition zone for the novel textile containing a poorly understood pathogenic fungus. The inhibition zone for the treated textiles varied from 11.7 ± 0.76 mm for L86 with *Fusarium proliferatum* to 22.0 ± 1.00 mm with *Aspergillus niger,* and from 19.0 ± 1.02 mm for LNP86 cloth with *Fusarium proliferatum* to 34.5 ± 2.18 mm with *Aspergillus flavus.* Data showed a significant increase for LNP86 textiles, from 38.4 to 36.2%. The treated textile by LNP 90 textile ranged between 21.8 ± 0.176 for LNP 90 textile with *Aspergillus niger* and 35.0 ± 2.00 mm with *Aspergillus ochraceus*, while the treated textile by L90 textile ranged between 15.0 ± 1.00 for L90 textile with *Aspergillus flavus* and 23.5 ± 0.71 mm with *Penicillium verrucosum*. According to the results, for the pathogenic fungus under study, the LNP 90 textile had a greater inhibition zone area than the L90 textile, increasing by 31.2 to 32.9%. The findings supported the antifungal finding made by Steeve et al*.*^62^ on lignin.

**Table 6:** Inhibition zone (mm) of LNP86, LNP90, L86, and L90 in the textile against pathogenic fungi.

Fungi	Inhibition zone (mean ± S.E.) (mm)					
	DMSO	Nystatin	LNP86	L86	LNP90	L90
*Aspergillus flavus*	0	29.67 ± 1.04^b^	34.5 ± 2.18^a^	12.6 ± 0.86^h^	29.5 ± 2.02^b^	15.0 ± 1.00^g^
*Aspergillus ochraceus*	0	30.50 ± .1.32^b^	31.5 ± 1.50^b^	14.5 ± 0.86^ g^	35.0 ± 2.00^a^	22.0 ± 1.58^e^
*Aspergillus niger*	0	32.17 ± 1.04^a^	25.0 ± 1.00^d^	22.0 ± 1.00^e^	21.8 ± 0.176^e^	22.3 ± 2.14^e^
*Fusarium proliferatum*	0	24.67 ± 1.04^c^	19.0 ± 1.02f	11.7 ± 0.76^i^	24.3 ± 1.58^cd^	18.0 ± 1.00f
*Penicillium verrucosum*	0	12.00 ± 0.50^d^	25.8 ± 1.28^c^	21.8 ± 1.08^e^	25.0 ± 1.40^c^	23.5 ± 0.71^d^
LSD (0.05)		1.35	0.72		1.02	

n = 3*S.E.: standard error, different subscripts are significantly different at the 5% level, negative control: DMSO, positive control: Nystatin.

The treated textile inhibition zone varied from 18.8 ± 0.58 for L86 textile with *Staphylococcus sciuri* to 28.7 ± 1.15 mm with *Escherichia coli* and from 22.3 ± 1.44 for LNP86 textile with *Staphylococcus sciuri* to 39.0 ± 1.73 mm with *Salmonella typhi,* according to previous findings on the inhibition zone for studied bacteria. As demonstrated in Table 7, the statistics showed a discernible increase for LNP86 textiles from 15.7 to 26.4%. The treated textile by LNP 90 textile varied between 23.0 ± 2.60 for LNP 90 textile with *Staphylococcus sciuri* and 38.3 ± 1.44 mm with *Salmonella typhi*, and between 22.0 ± 1.32 for L90 textile with *Staphylococcus aureus*. The data showed that for the studied pathogenic bacteria, LNP90 textile increased by 4.3 to 22.2%. All samples significantly affected the gram-negative bacteria types. Gram-positive bacteria’s L86 textile inhibition zone ranged from 18.8 to 20.3 mm, whereas gram-negative bacteria's inhibition zone ranged from 26 to 29 mm, with the inhibition zone for gram-negative bacteria increasing from 29.9 to 31.9%. More so, the LNP86 textile inhibition zone for gram-positive bacteria was 28.7 ± 1.15 to 24.7 ± 2.88 mm, and the zone for gram-negative bacteria was 31.3 ± 0.58 to 39.0 ± 1.73 mm. The inhibition zone for gram-negative bacteria went from 26.8 to 36.7%.

**Table 7:** Inhibition zone (mm) of LNP86, LNP90, L86, and L90 in the textile against pathogenic bacteria.

Bacteria	DMSO	Tetracycline	Inhibition zone (mean ± S.E.) (mm)			
			LNP86	L86	LNP90	L90
*Bacillus cereus*	0	15.2 ± 1.01^e^	24.7 ± 2.88^ef^	20.3 ± 0.58^h^	24.7 ± 1.15^g^	23.7 ± 1.15^h^
*Staphylococcus aureus*	0	20.2 ± 2.02^d^	24.2 ± 2.58f	19.3 ± 1.89^hi^	23.3 ± 2.52^h^	22.0 ± 1.32^i^
*Staphylococcus sciuri*	0	17.9 ± 1.29^d^	22.3 ± 1.44^g^	18.8 ± 0.58^i^	23.0 ± 2.60^h^	23.7 ± 1.15^h^
*Escherichia coli*	0	35.5 ± 2.18^a^	32.2 ± 1.15^c^	28.7 ± 1.15^d^	30.7 ± 1.15^c^	29.3 ± 1.53^e^
*Salmonella typhi*	0	32.2 ± 0.76^b^	39.0 ± 1.73^a^	28.0 ± 2.60^d^	38.3 ± 1.44^a^	29.8 ± 1.44^de^
*Salmonella enterica*	0	30.0 ± 1.32^b^	31.3 ± 0.58^c^	24.5 ± 0.86f	30.3 ± 1.44^cd^	26.8 ± 1.44f
*Pseudomonas aeruginosa*	0	25.5 ± 2.18^c^	33.7 ± 1.15^b^	26.0 ± 1.73^e^	36.5 ± 0.87^b^	27.5 ± 2.18f
LSD (0.05)		2.53	1.30		0.89	

n = 3, *S.E.: standard error, different subscripts are significantly different at the 5% level, negative control: DMSO, positive control: Tetracycline.

L90 textile samples had a higher effect than L86 on gram-positive and gram-negative bacteria, and their average was higher by 15.6 and 5.6%, respectively. Whereas the LNP86 sample average was higher than the LNP90 average by 7.5%, both LNP86 samples and LNP90 had the same zone area average with pathogenic positive bacteria. L90 exhibitsed more activity than L686 due to the presence of active groups like phenolic O–H, O-CH_3_, and C=C, which are responsible for the antimicrobial activity. The results were in agreement with those found by Poletto and Zattera^42^ and Ponomarenko et al*.*^43^. Furthermore, findings corroborated those of Dong et al*.*^52^, who discovered that lignin inhibited the growth of gram-positive bacteria (*Staphylococcus aureus*), and Yang et al*.*^63^, who mentioned that lignin has antioxidant, antifungal, and antiparasitic properties. According to Núñezflores et al*.*^64^, lignin can be used as a possible antioxidant and antibacterial agent and possesses antimicrobial effects against *Bacillus aryabhattai* and *Klebsiella sp.*

## Conclusion

The current study demonstrated the role of lignin and nano-lignins Giza 86 and 90 in expressing antioxidant and antimicrobial activity. When isolated using the alkaline method, lignin and nano-lignin, particularly in their nanosized form, are considered promising green agents. The Giza 90 cotton stalk cultivar was the best choice for obtaining antimicrobial lignin. This enhanced the quality of antimicrobial textiles and made them comparable to Nystatin and Tetracycline. Their bioactivity can help reduce environmental issues associated with the use of chemical materials. Therefore, isolating lignin using the alkaline method and preparing nano-lignin from Egyptian cotton stalks is a viable option for both environmental and economic applications, as opposed to burning it. So, it serves as a creative material for practical, economical, and environmental applications, including single-use clinical materials like gauze, clothing, bed sheets, and towels. This is particularly beneficial for patients who are not tolerant of antibiotics, such as those with diabetes, high blood pressure, and renal failure. The results show promise, indicating the need for further investigation using L90 and LNP90 as antitumor or antimicrobials against other pathogens.

## Data Availability

Data is provided within the manuscript.

## Abbreviations

DPPH: 2,2-Diphenyl-1-picrylhydrazyl radicals
FT-IR: Fourier transform infrared spectroscopy
L86: Lignin Giza86
L90: Lignin Giza90
LNP86: Lignin nanoparticles Giza86
LNP90: Lignin nanoparticles Giza90
MIC: Minimum inhibitory concentration
PDA: Potato dextrose agar
PS: Particle size
RCBD: Randomized complete block design
TEM: Transmission electron microscopy
TSB: Tryptic soy broth
ZP: Zeta potential
